# Increasing the CpG dinucleotide abundance in the HIV-1 genomic RNA inhibits viral replication

**DOI:** 10.1186/s12977-017-0374-1

**Published:** 2017-11-09

**Authors:** Irati Antzin-Anduetza, Charlotte Mahiet, Luke A. Granger, Charlotte Odendall, Chad M. Swanson

**Affiliations:** Department of Infectious Diseases, King’s College London, 3rd Floor Borough Wing, Guy’s Hospital, London, SE1 9RT UK

**Keywords:** HIV-1, Genomic RNA, CpG dinucleotide, Viral replication

## Abstract

**Background:**

The human immunodeficiency virus type 1 (HIV-1) structural protein Gag is necessary and sufficient to form viral particles. In addition to encoding the amino acid sequence for Gag, the underlying RNA sequence could encode *cis*-acting elements or nucleotide biases that are necessary for viral replication. Furthermore, RNA sequences that inhibit viral replication could be suppressed in *gag*. However, the functional relevance of RNA elements and nucleotide biases that promote or repress HIV-1 replication remain poorly understood.

**Results:**

To characterize if the RNA sequence in *gag* controls HIV-1 replication, the matrix (MA) region was codon modified, allowing the RNA sequence to be altered without affecting the protein sequence. Codon modification of nucleotides (nt) 22-261 or 22-378 in *gag* inhibited viral replication by decreasing genomic RNA (gRNA) abundance, gRNA stability, Gag expression, virion production and infectivity. Comparing the effect of these point mutations to deletions of the same region revealed that the mutations inhibited infectious virus production while the deletions did not. This demonstrated that codon modification introduced inhibitory sequences. There is a much lower than expected frequency of CpG dinucleotides in HIV-1 and codon modification introduced a substantial increase in CpG abundance. To determine if they are necessary for inhibition of HIV-1 replication, codons introducing CpG dinucleotides were mutated back to the wild type codon, which restored efficient Gag expression and infectious virion production. To determine if they are sufficient to inhibit viral replication, CpG dinucleotides were inserted into *gag* in the absence of other changes. The increased CpG dinucleotide content decreased HIV-1 infectivity and viral replication.

**Conclusions:**

The HIV-1 RNA sequence contains low abundance of CpG dinucleotides. Increasing the abundance of CpG dinucleotides inhibits multiple steps of the viral life cycle, providing a functional explanation for why CpG dinucleotides are suppressed in HIV-1.

**Electronic supplementary material:**

The online version of this article (10.1186/s12977-017-0374-1) contains supplementary material, which is available to authorized users.

## Background

The HIV-1 genomic RNA (gRNA) has three major functions in the viral life cycle [1]. First, it serves as the pre-mRNA that is spliced into over 70 different transcripts [2–4]. Second, it acts as the mRNA for the Gag and Gag-Pol polyproteins that comprise the structural and enzymatic proteins, respectively [5, 6]. Third, it is the genome that is packaged into virions and is reverse-transcribed upon infection of a new target cell [7, 8]. The gRNA can be divided into three regions: a 336 nt 5′ untranslated region (UTR), a 219 nt 3′ UTR, and an 8618 nt region that is densely packed with multiple open reading frames (nt lengths reference the HIV-1_NL4-3_ strain [9]). The 5′ UTR contains several *cis*-acting elements in complex stem-loop structures that regulate multiple stages of the viral life cycle including transcription, splicing, gRNA dimerization, encapsidation and reverse transcription [8, 10]. The central 8618 nt region encodes nine open reading frames: *gag, pol*, *vif*, *vpr*, *tat*, *rev*, *vpu*, *env* and *nef*.

In addition to encoding the amino acids of the viral proteins, the RNA sequence underlying the open reading frames could regulate multiple steps of the HIV-1 life cycle including splicing, RNA stability, RNA nuclear export, translation and reverse transcription. Indeed, a large number of *cis*-acting RNA elements within the protein coding regions have been reported to regulate HIV-1 replication, some of which are highly conserved and under purifying selection [11, 12]. These include the programmed ribosomal frameshift sequence in *gag* for Pol translation [13], splicing signals in *pol*, *vif*, *vpr*, *tat*, *rev* and *env* [2, 3], the Rev-response element (RRE) in *env* [14] and the polypurine tracts in *pol* and *nef* that are necessary for reverse transcription [15]. There is also extensive secondary and tertiary RNA structure throughout the gRNA that could regulate viral replication [16–19].

Determining the full complement of *cis*-acting elements in the gRNA that regulate viral replication is necessary for a complete understanding of the HIV-1 replication cycle and may aid in the development of novel antiviral therapies [20]. Furthermore, identifying and characterizing evolutionarily conserved *cis*-acting elements and structures is essential for understanding HIV-1 purifying and positive selection as well as recombination events [11, 12, 21–24]. Gag consists of four protein domains and two spacer peptides that control virion assembly [25]. Matrix (MA/p17) mediates Gag trafficking to the plasma membrane, capsid (CA/p24) forms the structure of the virion core, nucleocapsid (NC/p7) binds the genomic RNA to mediate encapsidation, and p6 recruits the ESCRT complexes necessary for membrane fission during budding. Within the MA open reading frame, there are a large number of proposed *cis*-acting RNA elements that could be necessary for viral replication. These include a hnRNPA1 binding site that may regulate gRNA nuclear export [26], an intronic splice enhancer [27, 28], an internal ribosome entry site [29], instability sequences that lead to RNA degradation in the absence of Rev [30], sequences that base pair with the 5′ and 3′ UTRs [31–35], and elements that regulate encapsidation [7, 8]. However, the functional relevance of most of these elements for viral replication is unclear.

Some nucleotide patterns may also regulate HIV-1 replication and be under evolutionary selection. For example, the base composition of HIV-1 deviates from that of the human genome. HIV-1 RNA has a high percentage of adenine (A, 36%) and low percentage of cytosine (C, 18%) [36–43]. This nucleotide bias is found in groups M, N and O and is a general property of lentiviruses, though not all retroviruses [36, 38, 42, 44]. Even though HIV-1 has a very high nucleotide substitution rate and sequence diversity, the A-rich bias has been conserved during the HIV-1 pandemic [42]. There are two hypotheses for why this has been maintained in the virus. First, the mutational pattern of reverse transcriptase or antiviral APOBEC3 proteins could impose an A-rich nucleotide bias [45–52]. Second, this bias could be required for viral replication and be under purifying selection [38, 53, 54].

In addition to understanding the RNA elements that are necessary for viral replication, it is important to characterize the motifs that are underrepresented and may be deleterious. HIV-1 has a much lower than expected frequency of the dinucleotide CpG [36, 40, 44, 55–57]. This has been proposed to be under negative selection and the CpG dinucleotide abundance in HIV-1 may be linked to disease progression [58]. However, the mechanism by which CpG dinucleotides affect viral replication is unknown.

These nucleotide biases cause the HIV-1 open reading frames to have a codon usage pattern that differs substantially from that of human mRNAs [36–41, 43]. The genetic code is redundant in that there are 61 codons for 20 amino acids and all of the amino acids except methionine and tryptophan are encoded by at least two codons. The preferred codons in cellular mRNAs are thought to correlate with the availability of the aminoacyl-tRNAs but HIV-1 contains many rare codons [36–41].

In this study, we investigated whether RNA elements in the MA region of *gag* positively or negatively regulate HIV-1 replication. We initially focused on this region because of its high content of potential RNA regulatory elements (discussed above). To change the RNA sequence without altering the amino acid sequence, we codon modified this region by introducing large numbers of synonymous mutations. These mutations strongly inhibited viral replication by decreasing gRNA abundance, gRNA stability, Gag expression, virion production and infectivity. We found that CpG dinucleotides introduced during codon modification were necessary and sufficient to attenuate HIV-1 replication. This highlights the functional importance of the suppressed CpG abundance in HIV-1 [36, 40, 44, 55–57] and shows that increasing the number of CpG dinucleotides in the gRNA inhibits multiple steps of the viral life cycle.

## Methods

### Cell culture and transfections

Jurkat cells were cultured in RPMI 1640 GlutaMAX Medium (Gibco) supplemented with 10% fetal bovine serum (FBS) and 1% penicillin–streptomycin. Hela, TZM-bl and 293T cells were cultured in Dulbecco’s Modified Eagle Medium (Gibco) supplemented with 10% FBS and 1% penicillin–streptomycin. All cell lines were grown at 37 °C in a humidified atmosphere with 5% CO_2_.

### Plasmids

The pHIV-1_NL4-3_ constructs used in this study contain the provirus sequence from pHIV-1_NL4-3_ [9] cloned into the KpnI and SalI sites of pGL4.10 (Promega). pHIV-1 CM22-261, pHIV-1 CM22-165, pHIV-1 CM166-261 and pHIV-1 CM22-378 have the designated sequences from pHDMHgpm2 [59] chemically synthesized by Life Technologies and cloned into pHIV-1_NL4-3_. For pHIV-1CM 22-261_lowCpG_ and pHIV-1 CM22-378_lowCpG_, pHIV-1 CpG22-261 and pHIV-1 CpG22-378, the sequences shown in Fig. 6 were synthesized by Life Technologies and cloned into pHIV-1_NL4-3_. pHIV-1 ∆22-261 and pHIV-1 ∆22-378 have the designated region in *gag* replaced with a XbaI site as in Reil et al. [60]. The modified sequences in these plasmids were verified by DNA sequencing (Eurofins). pGFP and pVSV-G have been previously described [61, 62].

### HIV-1 spreading infection assay

4 × 10^6^ 293T cells were seeded in 10 cm plates and transfected with 10 μg of pHIV-1 and 1.25 μg of pGFP using poly(ethlyleneimine) solution (PEI) at a ratio of 5 μl PEI per 1 μg DNA. Approximately 48-h post-transfection, the media was harvested, filtered through a 0.45 μm filter and quantified using a p24^Gag^ enzyme-linked immunosorbent assay (ELISA) (Perkin-Elmer). A total of 2.5 × 10^5^ Jurkat cells were plated in 1 mL of medium per well in 48 well plates and infected with 25 ng of p24^Gag^ of each virus. SupT1 cells were infected with 10 ng of p24^Gag^ for each virus. Supernatants were first collected when syncytia were first observed in the culture infected with HIV-1_NL4-3_. The amount of infectious virus present at each time point was quantified by infecting the TZM-bl indicator cell line [63–65]. Infectivity was measured by the induction of β-galactosidase using the Galacto-Star™ System (Applied Biosystems).

### Single cycle infectivity assay

Six-well plates of HeLa cells were transfected using TransIT^®^-LT1 (Mirus) according to the manufacturer’s instructions at the ratio of 3 μL TransIT^®^-LT1 to 1 μg DNA. For each transfection, 0.5 μg pHIV-1 and 0.5 μg pGFP or pVSV-G was used. Media was recovered approximately 48 h post-transfection and filtered through a 20% sucrose cushion for 2 h at 20,000×*g*. The amount of infectious virus was quantified by using the TZM-bl indicator cell line [63–65].

### Analysis of protein expression by immunoblotting

Approximately 48-h post-transfection, HeLa cells were lysed in radioimmunoprecipitation assay (RIPA) buffer (10 mM Tris–HCl, pH 7.5, 150 mM NaCl, 1 mM EDTA, 0.1% SDS, 1% Triton X-100, 1% sodium deoxycholate). The media was clarified using a 0.45 μm filter. Virions were pelleted through a 20% sucrose cushion in phosphate-buffered saline (PBS) solution for 2 h at 20,000×*g*. The pellet was resuspended in 2× loading buffer (60 mM Tris–HCl (pH 6.8), 10% β-mercaptoethanol, 10% glycerol, 2% sodium dodecyl sulfate (SDS), 0.1% bromophenol blue). Cell lysates and virions were resolved by SDS–polyacrylamide gel electrophoresis and transferred to a nitrocellulose membrane. The primary antibodies used were specific to HIV-1 p24^Gag^ [66], Hsp90 (sc7947: Santa Cruz Biotechnology), phosphoSTAT1 (612132: BD Transduction), IFIT1 (GTX118713-S: Insight Biotechnology) or β-actin (ac-15: Sigma). Dylight™ 800-conjugated secondary antibodies (5151S and 5257S: Cell Signaling) were used to detect the bound primary antibodies with the Li-CoR infrared imaging (LI-COR UK LTD).

### Quantitative RT-PCR

Hela cells were washed with 1xPBS and the RNA was extracted using the RNeasy kit (Qiagen) following the manufacturer’s instructions. 1 μg of RNA was reverse transcribed to cDNA using the High Capacity cDNA archive kit (Applied Biosystems). RNA from virions was isolated using QIAamp viral RNA mini kit following the manufacturer’s instructions. Because carrier RNA is added to the lysis buffer, the total RNA isolated was quantified using a Qubit 3.0 fluorometer (ThermoFisher) and normalized so that 20 ng of RNA from each sample was reverse transcribed using the High Capacity cDNA archive kit (Applied Biosystems). PCR reactions were performed in triplicate with Taqman Universal PCR mix using the Applied Biosystems 7500 real-time PCR system. HIV-1_NL4-3_ gRNA primers were GGCCAGGGAATTTTCTTCAGA/TTGTCTCTTCCCCAAACCTGA (forward/reverse) and the probe was FAM-ACCAGAGCCAACAGCCCCACCAGA-TAMRA. HIV-1_NL4-3_ total RNA primers were TAACTAGGGAACCCACTGC/GCTAGAGATTTTCCACACTG (forward/reverse) and the probe was FAM-ACACAACAGACGGGCACACACTA-TAMRA. To analyze gRNA stability, 1 µg/ml Actinomycin D (Sigma Aldrich) was added to HeLa cells ~ 45 h post-transfection. RNA was isolated at the designated timepoints and gRNA abundance was measured.

### TLR and IFN stimulations, Sendai virus infection

HeLa cells were stimulated with synthetic TLR ligands for 5 h at the concentrations indicated. Ligands supplied by Invivogen were polyIC: polyIC (tlrl-pic), Gardiquimod (tlrl-gdqs), CL075 (tlrl-c75), R848 (tlrl-r848), Pam3CSK4 (P3C. tlrl-pms), Ultrapure Flagellin (FliC-tlrl-epstfla-5). LPS was supplied by Enzo (ALX-581-012-L002). CpG DNA was synthesised by IDT and 23S ribosomal RNA by Sigma. Sendai Virus (SeV) was obtained from Charles River labs. IFN-β was purchased from Peprotech and was added to the culture for 1 h to activate IFN signaling.

### Sequence analysis of the HIV-1_NL4-3_ gRNA

The “analyze base composition” tool in MacVector was used to calculate the mono- and di-nucleotide frequencies for the HIV-1_NL4-3_ gRNA (NCBI accession number M19921). The dinucleotide frequencies are calculated using the following formula: number of dinucleotide occurrences/(frequency of base 1 in pair × frequency of base 2 in pair) where frequency of base is number of occurrences of base/total number of bases in sequence. WebLogo [67] was used to generate conserved nucleotides surrounding the CpG dinucleotides.

## Results

### Synonymous mutations in *gag* inhibit HIV-1 replication

**Fig. 1:**
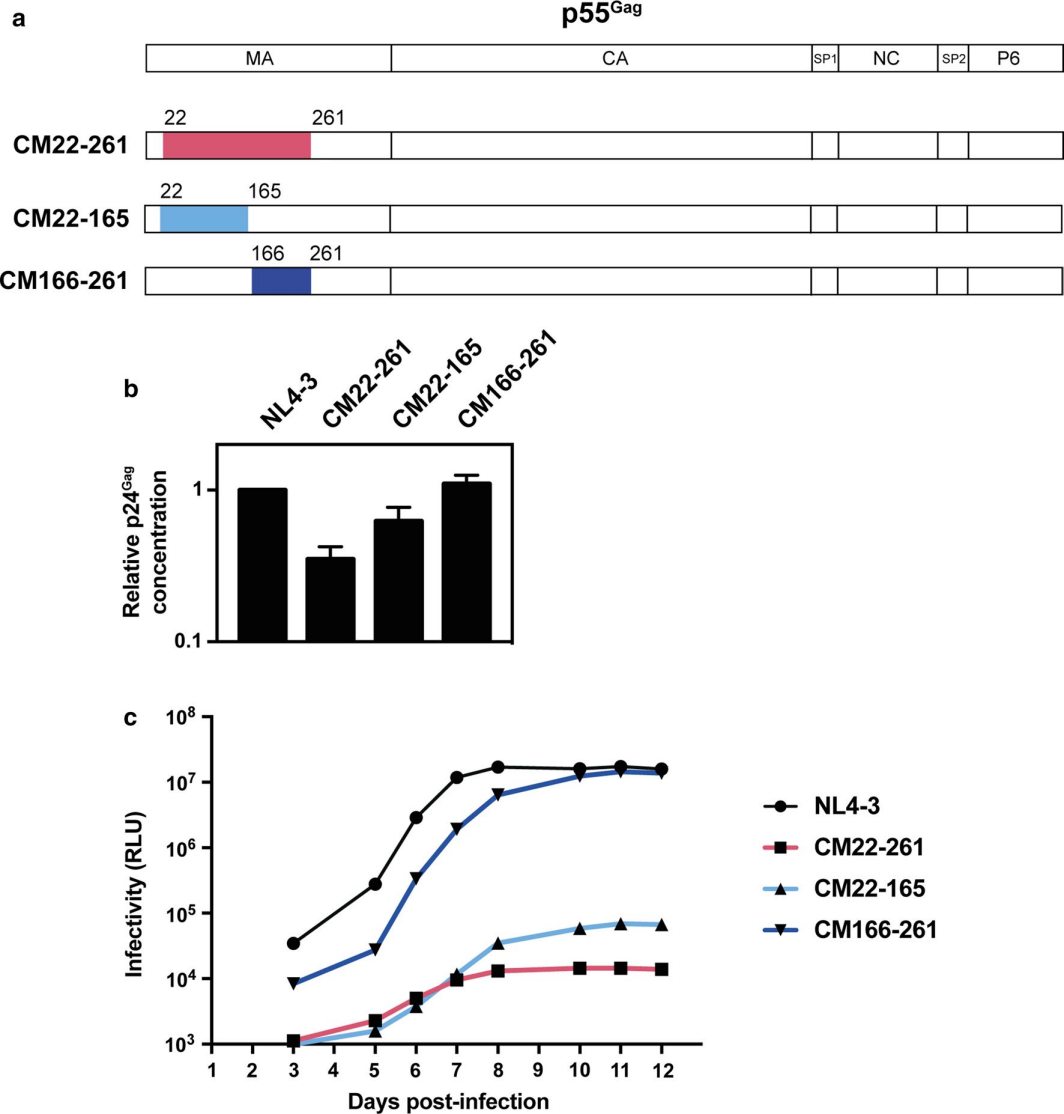
Codon modification of nucleotides 22-261 in *gag* inhibits viral replication. **a** Schematic representation of p55^Gag^ in HIV-1_NL4-3_, HIV-1 CM22-261, HIV-1 CM22-165 and HIV-1 CM166-261. **b** The amount of HIV-1 CA (p24^Gag^) in supernatants from 293T cells transfected with pHIV-1_NL4-3_, pHIV-1 CM22-261, pHIV-1 CM22-165 or pHIV-1 CM166-261 were quantified by p24^Gag^ ELISA. The bar chart is the average of three independent experiments normalized to HIV-1_NL4-3_. Error bars represent standard deviation. **c** Jurkat cells were infected with 25 ng of p24^Gag^ for each indicated virus. The amount of infectious virus present at each time point was measured in TZM-bl cells. This is representative of three independent experiments

To analyze the functional relevance of RNA elements and nucleotide bias underlying the MA domain in Gag, we introduced 80 synonymous mutations into nt 22-261 of HIV-1_NL4-3_ *gag* (Fig. 1a). This codon modified (CM) provirus, HIV-1 CM22-261, has 69/80 codons in this region altered without affecting the amino acid sequence. The mutations were derived from pHDMHgpm2, a codon optimized Gag-Pol construct in which many of the HIV-1 codons are replaced with codons used in highly expressed human mRNAs [59, 68]. In addition, nt 22-165 or 166-261 in *gag* were codon modified to produce HIV-1 CM22-165 and HIV-1 CM166-216, which have 49 and 31 synonymous mutations, respectively. Virus stocks were prepared by transfecting 293T cells with each proviral DNA construct and the concentration of viral CA/p24^Gag^ for each stock was measured by ELISA. HIV-1 CM22-261 and HIV-1 CM22-165 had an ~ 65% and ~ 40% decrease in p24^Gag^ concentration, respectively (Fig. 1b). To analyze the fitness of each virus, the viral inoculum was normalized so that Jurkat CD4 T cells were challenged with 25 ng of p24^Gag^ for each virus (Fig. 1c). The amount of infectious virus in the culture supernatant was monitored over 2 weeks using TZM-bl indicator cells [63–65]. HIV-1 CM22-261 replicated at a very low but detectable level and at day 12 had > 99.9% less infectivity than wild type virus. HIV-1 CM22-165 replicated slightly better than HIV-1 CM22-261, but was still > 99% lower than wild type HIV-1 at day 12. HIV-1 CM166-261 plateaued at the same level as wild type HIV-1 but with a delay of ~ 3 days. Similar results were observed when SupT1 CD4 T cells were challenged with the wild type and mutated viruses (Additional file 1).

**Fig. 2:**
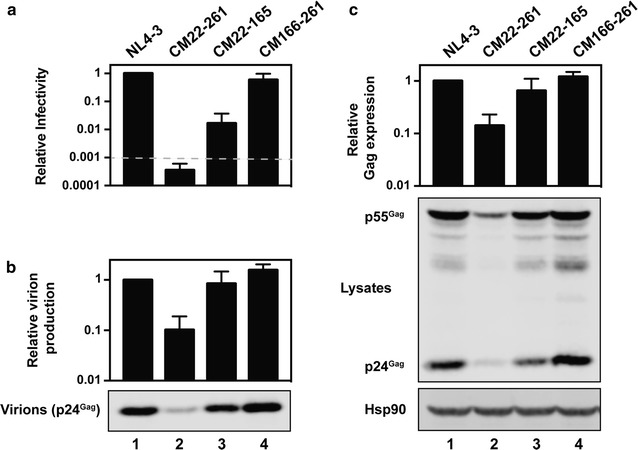
Codon modification of nucleotides 22-261 in *gag* inhibits infectious virus production. HeLa cells were transfected with pHIV-1_NL4-3_, pHIV-1 CM22-261, pHIV-1 CM22-165 or pHIV-1 CM166-261. **a** Culture supernatants were used to infect TZM-bl reporter cells to measure viral infectivity. The bar charts show the average values of six independent experiments normalized to the value obtained for HIV-1_NL4-3_. The average relative light units (RLU) for HIV-1_NL4-3_ is 3,680,747. The dashed line represents 1000 RLU, which is approximately the limit of the assay for reproducible differential results. Error bars represent standard deviation. **b**, **c** Gag expression in the media (**b**) and cell lysate (**c**) was detected using quantitative immunoblotting. The bar charts show the average of four independent experiments normalized to HIV-1_NL4-3_. Error bars represent standard deviation

We then used a single cycle infectivity assay to determine if HeLa cells were non-permissive for HIV-1 CM22-261, HIV CM22-165 or HIV-1 CM166-261 replication as well as to characterize which steps in the viral life cycle are inhibited by the synonymous changes in *gag*. HeLa cells were transfected with pHIV-1_NL4-3_, pHIV-1 CM22-261, pHIV-1 CM22-165 or pHIV-1 CM166-261 and the media and cell lysates were harvested ~ 48 h later. HIV-1 infectivity in the media was determined using TZM-bl cells and the abundance of virions in the media and Gag in the cell lysate was analyzed by quantitative immunoblotting. Compared with the wild type virus, HIV-1 CM22-261 infectivity was decreased to the limit of detection of the assay (Fig. 2a), indicating that the virus is attenuated in HeLa cells. Virion production and intracellular Gag expression were decreased ~ 90% (Fig. 2b, c). For HIV-1 CM22-165, the amount of infectious virus in the media was decreased ~ 98% with a < 50% decrease in Gag expression and viron production. HIV-1 CM166-261 consistently yielded similar amounts of infectivity, virions and intracellular Gag expression as wild type HIV-1. Overall, there is a substantial reduction in infectivity for HIV-1 CM22-261 and HIV-1 CM22-165. HIV-1 CM22-261 also has a substantial defect in Gag expression and virion production.

**Fig. 3:**
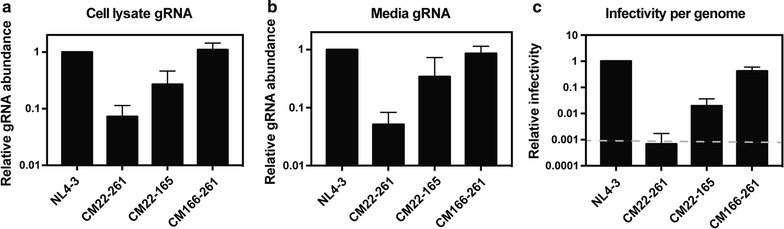
Codon modification of nucleotides 22-261 in *gag* decreases gRNA abundance. HeLa cells were transfected with pHIV-1_NL4-3_, pHIV-1 CM22-261, pHIV-1 CM22-165 or pHIV-1 CM166-261. The bar charts show the average values of three independent experiments normalized to HIV-1_NL4-3_. Error bars represent standard deviation. **a** RNA was extracted from cell lysates and gRNA abundance was quantified by qRT-PCR. **b** RNA was extracted from the media and gRNA abundance was quantified by qRT-PCR. **c** Equivalent amounts of HIV-1 genomes were used to infect TZM-bl reporter cells to measure infectivity. The bar charts show the average values of three independent experiments normalized to the value obtained for HIV-1_NL4-3_. The average RLU for HIV-1_NL4-3_ is 1,146,196. The dashed line represents 1000 RLU, which is approximately the limit of the assay for reproducible differential results

To determine if the decrease in infectious virus production was due to a decrease in gRNA abundance, we performed quantitative RT-PCR (qRT-PCR) using a primer–probe set in a region of *gag* that was not mutated (Fig. 3a, b). pHIV-1_NL4-3_, pHIV-1 CM22-261, pHIV-1 CM22-165 and pHIV-1 CM22166-261 were transfected into HeLa cells. RNA was isolated from the cell lysate and media ~ 48 h post-transfection. HIV-1 CM22-261 gRNA was reduced > 90% in the cell lysate and media compared with the wild type virus. HIV-1 CM22-165 gRNA was decreased ~ 70% in the cell and ~ 65% in the media. HIV-1 CM166-261 gRNA abundance was equivalent to wild type HIV-1 in the cell lysate and media. We then determined the effect of the synonymous mutations on infectivity/viral genome by infecting TZM-bl cells with an equivalent amount of viral genomes for each virus. When the input number of genomes was normalized based on the results in Fig. 3b, HIV-1 CM22-261 infectivity was at the limit of detection of the assay and HIV-1 CM22-165 infectivity was decreased ~ 98% (Fig. 3c). This indicates that the decreased abundance of viral genomes in the media is not fully responsible for the loss of infectivity for HIV-1 CM22-261 and HIV-1 CM22-165.

The HIV-1 gRNA can be spliced into over 70 different transcripts [4] and the *gag*-*pol* intron can be spliced out through one 5′ splice site and six 3′ splice sites [2]. A potential explanation for the decrease in intracellular gRNA abundance is that the mutations in *gag* could affect intronic splicing silencer (ISS) sequences. If this occurred, gRNA abundance would decrease due to oversplicing but the total amount of HIV-1 RNA would stay the same. To test this, we determined the total intracellular HIV-1 RNA abundance using a primer–probe set upstream of the major 5′ splice donor (SD1). HIV-1 CM22-261 and HIV-1 CM22-165 had an ~ 80 and ~ 60% decrease in total HIV-1 RNA abundance (Additional file 2). Since ~ 50% of the gRNA remains unspliced [2], this is consistent with a specific reduction in the gRNA and does not appear to be a consequence of oversplicing.

### CpG dinucleotides are necessary for the inhibition of infectious virus production

The synonymous mutations introduced into *gag* could inhibit HIV-1 replication by inactivating essential *cis*-acting RNA elements or introducing inhibitory elements. The region mutated in HIV-1 CM22-261 was designed to match the codons previously deleted by Reil et al. [60] in HIV-1_HXBH10_∆8-87/∆CT. In this virus, amino acids 8-87 (nt 22-261) in Gag were deleted and a stop codon in Env removed the cytoplasmic tail domain. Because deletions in the globular core domain of MA prevent incorporation of Env with a full cytoplasmic domain [69], the truncated Env cytoplasmic tail is necessary for virion infectivity. However, pseudotyping with heterologous envelope glycoproteins, such as that from vesicular stomatitis virus (VSV-G), allow viral entry into a target cell. HIV-1_HXBH10_ ∆8-87/∆CT replicates as well as HIV-1_HXBH10_ ∆CT in the MT4 cell line [60], indicating that neither the protein or RNA sequences in this region are necessary for viral replication in these cells.

**Fig. 4:**
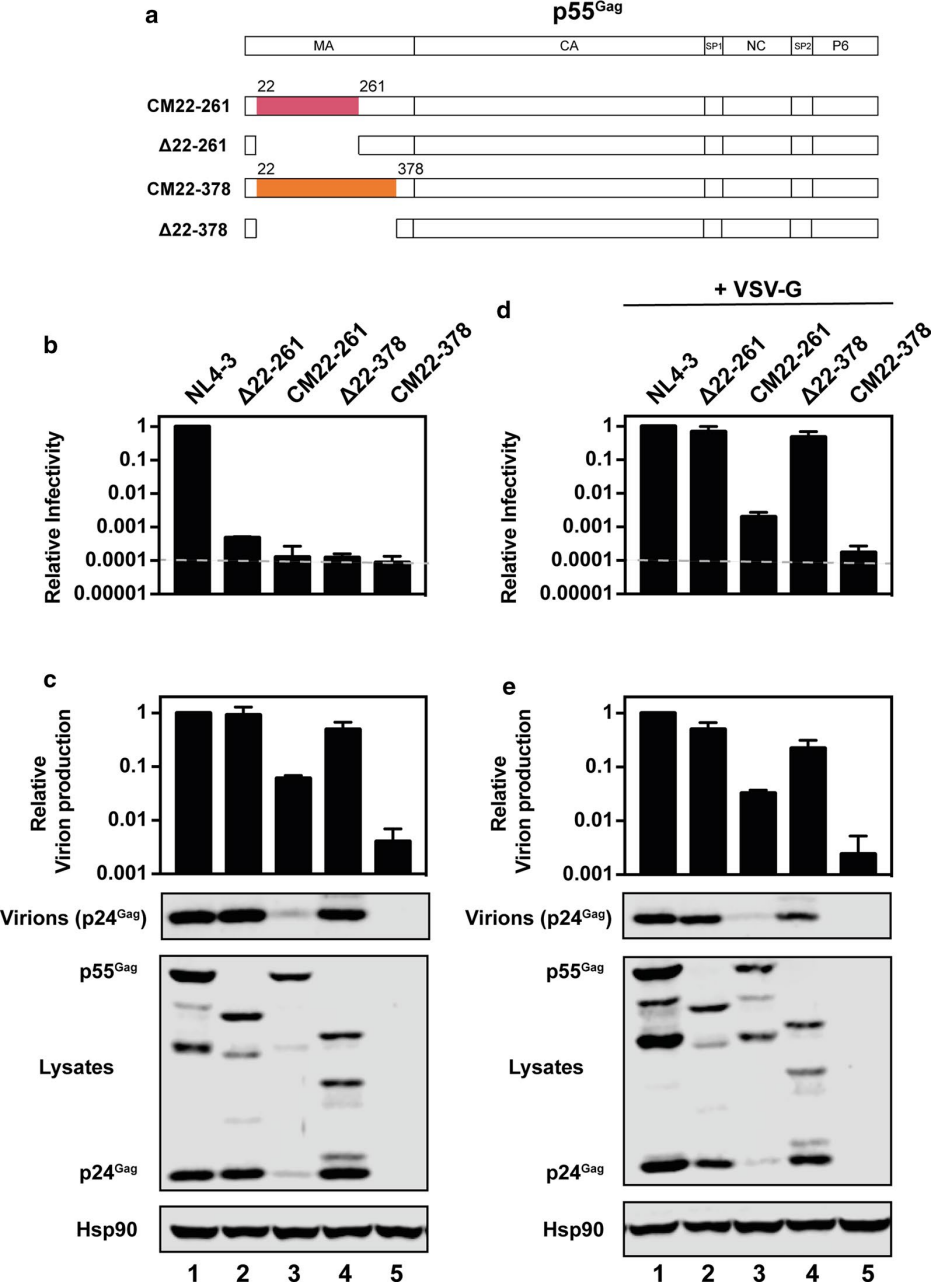
Codon modification but not deletion of nucleotides 22-261 or 22-378 in *gag* inhibits infectious virus production. **a** Schematic representation of p55^Gag^ in HIV-1_NL4-3_, HIV-1 CM22-261, HIV-1 Δ22-261, HIV-1 CM22-378 or HIV-1 Δ22-378. **b**, **d** HeLa cells were transfected with pHIV-1_NL4-3_, pHIV-1 CM22-261, pHIV-1 Δ22-261, pHIV-1 CM22-378 or pHIV-1 Δ22-378 and pGFP (**b**) or pVSV-G (**d**). The amount of infectious virus in the culture supernatants was measured in TZM-bl cells. The average RLU for HIV-1_NL4-3_ + GFP and HIV-1_NL4-3_ + VSV-G is 13,701,427 and 16,981,387, respectively. The dashed line represents 1000 RLU, which is approximately the limit of the assay for reproducible differential results. **c**, **e** Gag expression in the media and cell lysate were measured by quantitative western blotting. **b**–**e** The bar charts show the average of three independent experiments relative to HIV-1_NL4-3_. Error bars represent standard deviation

To determine whether the synonymous mutations inserted into nt 22-261 of *gag* removed essential *cis*-acting elements or inserted deleterious sequences, we generated a HIV-1_NL4-3_ ∆22-261 provirus construct (Fig. 4a) and compared it with HIV-1 CM22-261 in the absence or presence of VSV-G. In the absence of VSV-G, HIV-1 ∆22-261 produced very low levels of infectious virus (Fig. 4b), which was expected due to the role of MA in recruiting Env with a full-length cytoplasmic tail. Gag expression and virion production were similar for wild type HIV-1 and HIV-1 ∆22-261 (Fig. 4c), indicating that RNA or protein sequences in this region are not necessary for these steps of the viral life cycle. When the viruses were pseudotyped with VSV-G, HIV-1 ∆22-261 infectivity was similar to wild type virus, confirming that the only functional defect for this virus in HeLa cells is Env incorporation. In contrast, HIV-1 CM22-261 was not rescued by VSV-G pseudotyping and had a > 99.9% reduction in infectivity (Fig. 4d).

Reil et al. [60] also deleted amino acids 8-126 (nt 22-378) in MA and found that HIV-1_HXBH10_ ∆8-126/∆CT replicated with moderately delayed kinetics compared to HIV-1_HXBH10_ ∆CT in MT4 cells. We produced HIV-1_NL4-3_ provirus constructs in which this region was either deleted (HIV-1 ∆22-378) or codon modified (HIV-1 CM22-378). VSV-G pseudotyped HIV-1 ∆22-378 had a small decrease in infectious virus production compared to wild type HIV-1 (Fig. 4d), which correlated with virion production (Fig. 4e). However, VSV-G pseudotyped HIV-1 CM22-378 had a > 99.9% decrease in infectious virus production with Gag expression and virion production near the limit of detection (Fig. 4d, e). Therefore, we concluded that codon modification of nt 22-261 or nt 22-378 of *gag* introduced inhibitory sequences into the HIV-1 genome that reduce Gag expression, virion production and infectivity.

**Fig. 5:**
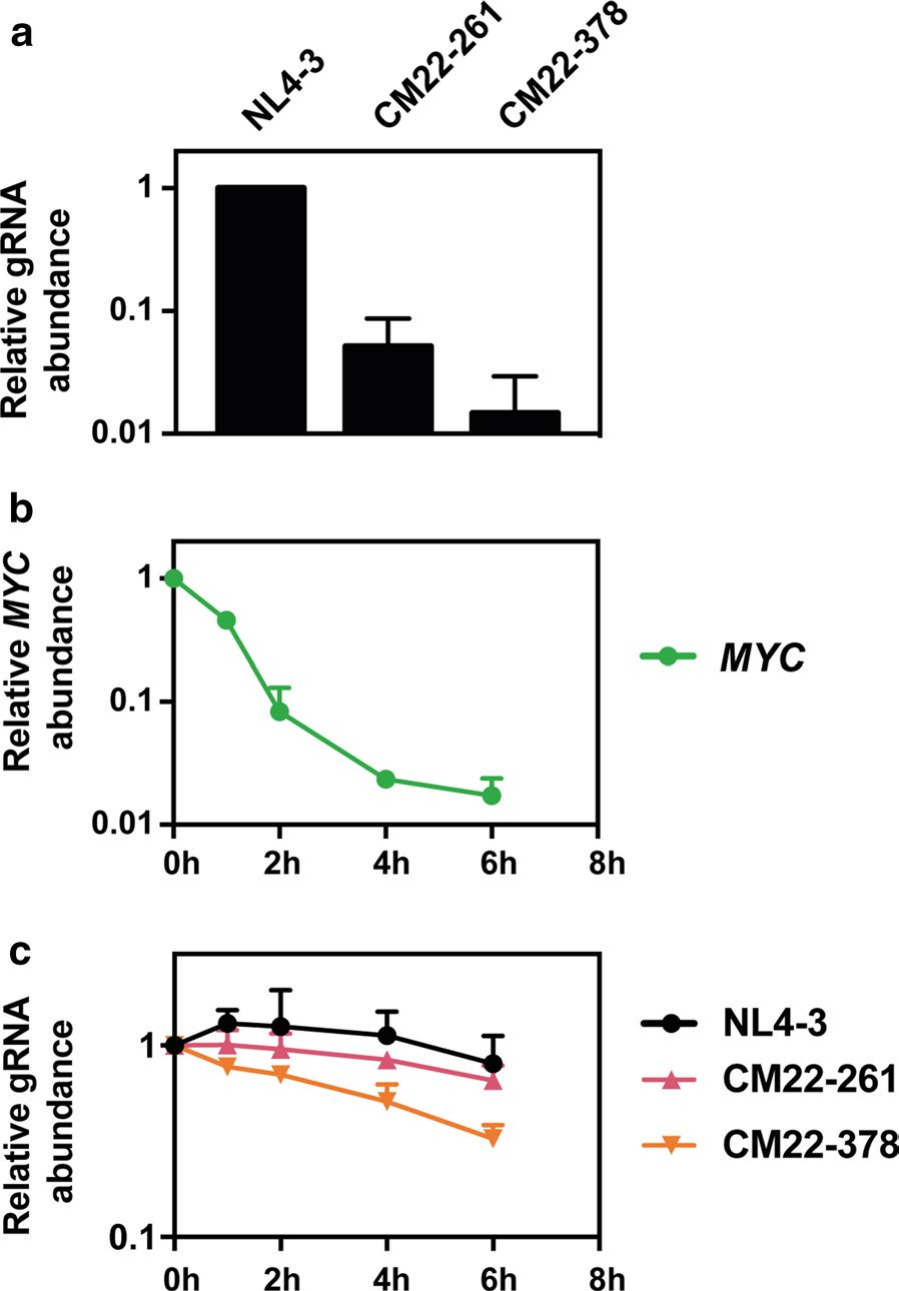
Codon modification of nucleotides 22-261 and 22-378 in *gag* decreases gRNA abundance and stability. HeLa cells were transfected with pHIV-1_NL4-3_, pHIV-1 CM22-261 or pHIV-1 CM22-378. **a** RNA was extracted from cell lysates at the 0 h timepoint and gRNA abundance was quantified by qRT-PCR. The bar charts show the average values of three independent experiments normalized to HIV-1_NL4-3_. Error bars represent standard deviation. **b**, **c** Actinomycin D was added to inhibit RNA polymerase II transcription and the abundance of *MYC* mRNA (**b**) or gRNA (**c**) was measured at 0, 1, 2, 4 and 6 h post-addition. Each value is relative to the 0 h timepoint and is an average of three independent experiments. Error bars represent standard deviation

To determine whether the synonymous mutations in *gag* altered the stability of the viral RNA, HeLa cells were transfected with pHIV-1_NL4-3_, pHIV-1 CM22-261 or pHIV-1 CM22-378 and, ~ 45 h post-transfection, RNA polymerase II-dependent transcription was inhibited by adding actinomycin D. RNA was isolated from the cells immediately before actinomycin D addition (0 h) and then 1, 2, 4 and 6 h thereafter. gRNA abundance at the 0 h timepoint was substantially decreased for HIV-1 CM22-261 and HIV-1 CM22-378 (Fig. 5a) and correlated with the length of codon modified sequence. Since *MYC* mRNA has a half-life of < 1 h [70], we used it as a control for RNA stability and analyzed its abundance at each timepoint. As expected, *MYC* mRNA was rapidly degraded (Fig. 5b). The gRNA abundance for HIV-1_NL4-3_, HIV-1 CM22-261 and HIV-1 CM22-378 decreased by ~ 20, ~ 35 and ~ 70%, respectively, at the 6 h timepoint relative to its abundance at 0 h (Fig. 5c). This indicates that HIV-1 CM22-261 and HIV-1 CM22-378 gRNA is less stable than HIV-1_NL4-3_ gRNA. Comparing HIV-1 CM22-378 gRNA abundance to that of HIV-1_NL4-3_ at the 6 h timepoint, HIV-1 CM22-378 gRNA was ~ 60% lower than wild type virus gRNA. If the degradation rate is constant, a 50% decrease every 6 h in gRNA abundance for HIV-1 CM22-378 relative to HIV-1_NL4-3_ would be compounded to yield a 98.4% decrease after 36 h. This is consistent with the ~ 98% decrease in steady state gRNA for HIV-1 CM22-378 that we observed ~ 45 h post-transfection (Fig. 5a). Overall, the synonymous mutations introduced into *gag* appear to decrease the stability of the gRNA.

Two types of RNA dinucleotide patterns have previously been implicated in restricting RNA virus replication, UpA and CpG [71–74]. The observed/expected ratio for UpA in the HIV-1_NL4-3_ gRNA is 0.92 (Table 1) and the total number of UpA dinucleotides in nt 22-261 and 22-378 decreased substantially in the codon modified sequence compared with the wild type sequence (Table 2). This indicates that UpA dinucleotide content is not causing the inhibitory phenotype. The CpG dinucleotide observed/expected ratio is 0.21 in HIV-1_NL4-3_ and it is the only dinucleotide substantially suppressed (Table 1). Within nt 22-261 of *gag*, wild type HIV-1 has 4 CpG dinucleotides and the codon modified sequence has 22 (Table 2). Similarly, codon modification of nt 22-378 increased the number of CpGs from 4 to 30. Because CpG dinucleotides are underrepresented in HIV-1 (Table 1) [40, 44, 55–57] and previous reports have shown that increasing the CpG dinucleotide abundance inhibits picornavirus and influenza virus replication [71–74], we hypothesized that the increased number of CpG dinucleotides in HIV-1 CM22-261 and HIV-1 CM22-378 could cause the decrease in HIV-1 infectious virion production.

**Table 1:** HIV-1_NL4-3_ genomic RNA mononucleotide and dinucleotide frequencies

Base	**#**	Freq.	%	Obs./Exp.
Mononucleotide frequencies				
A	3281	0.358	35.8	1.43
C	1635	0.178	17.8	0.71
G	2216	0.242	24.2	0.97
T	2041	0.223	22.3	0.89
G + C	3851	0.420	42.0	0.84
A + T	5322	0.580	58.0	1.16
Total positions = 9173				

Base	**#**	Freq.	%	Obs./Exp.
Dinucleotide frequencies				
AA	1093	0.119	11.9	0.93
AC	522	0.057	5.7	0.89
AG	962	0.105	10.5	1.21
AT	703	0.077	7.7	0.96
CA	758	0.083	8.3	1.30
CC	371	0.040	4.0	1.27
CG	82	0.009	0.9	0.21
CT	424	0.046	4.6	1.17
GA	762	0.083	8.3	0.96
GC	424	0.046	4.6	1.07
GG	625	0.068	6.8	1.17
GT	405	0.044	4.4	0.82
TA	668	0.073	7.3	0.92
TC	318	0.035	3.5	0.87
TG	546	0.060	6.0	1.11
TT	509	0.055	5.5	1.12
Total positions = 9172

**Table 2:** Changes in nucleotide composition and total number of mutations for codon modification of regions 22-261 and 22-378 in *gag*

Construct	% A	% C	% G	% T	Total UpA	Total CpG	Total number mutations relative to wild type
WT 22-261	38	17	23	22	23	4	
CM 22-261	18	36	32	15	3	22	80
CM 22-261_lowCpG_	24	27	34	15	5	4	59
CpG 22-261	32	25	22	21	19	22	21
WT 22-378	42	17	24	18	29	4	
CM 22-378	22	35	32	12	3	30	109
CM 22-378_lowCpG_	28	26	33	13	7	4	79
CpG 22-378	36	25	22	17	23	30	30

**Fig. 6:**
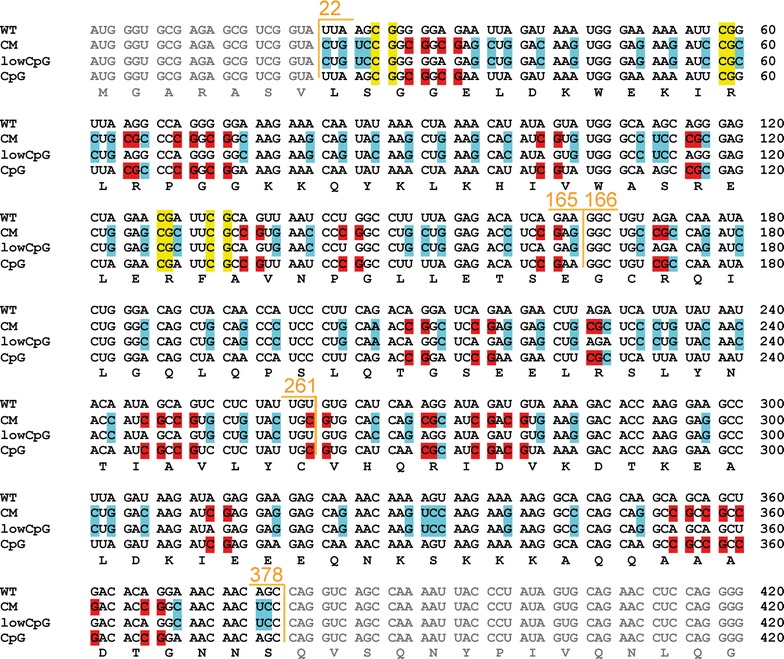
Schematic representation of nucleotides 1-420 in *gag* for HIV-1_NL4-3_, HIV-1 CM22-261, HIV-1 CM22-165, HIV-1 CM166-261, HIV-1CM22-261_lowCpG_, HIV-1 CM22-378 and HIV-1 CM22-378_lowCpG_. Mutations that are the result of codon modification and do not introduce CpG dinucleotides are highlighted in turquoise. CpG dinucleotides present in HIV-1_NL4-3_ are highlighted in yellow and CpG dinucleotides introduced by codon modification are highlighted in red

**Fig. 7:**
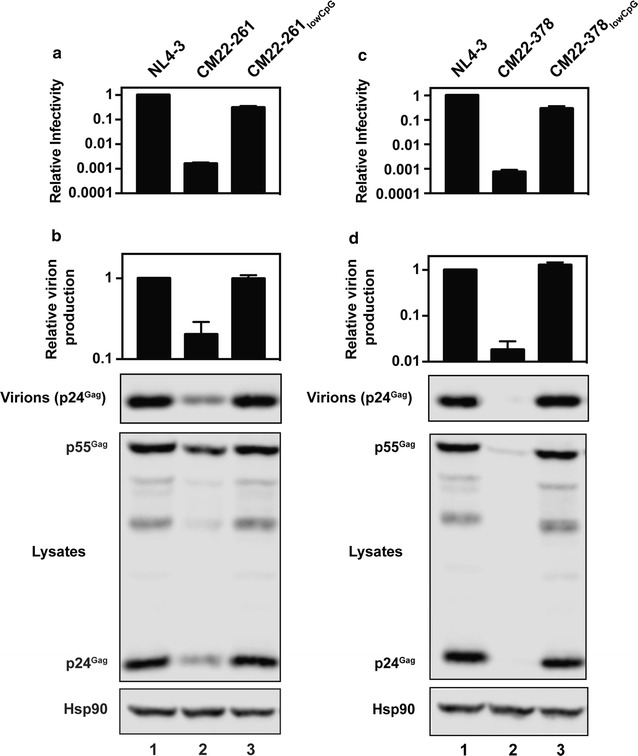
Decreasing the CpG abundance within HIV-1 CM22-261 and HIV-1 CM22-378 restores infectious virus production. HeLa cells were transfected with pHIV-1_NL4-3_, pHIV-1 CM22-261, pHIV-1 CM22-261_lowCpG_, pHIV-1 CM22-378 and pHIV-1 CM22-378_lowCpG_. The bar charts show the average of three independent experiments relative to HIV-1_NL4-3_. Error bars represent standard deviation. **a**, **c** The amount of infectious virus present in the media was measured in TZM-bl cells. **b**, **d** Virion production and intracellular Gag expression was measured using quantitative western blotting

To test this hypothesis, we synthesized a HIV-1 *gag* sequence containing all of the synonymous mutations present in pHIV CM22-261 with the exception of the codon changes that introduced CpG dinucleotides (Fig. 6) and inserted it into pHIV_NL4-3_ to produce pHIV-1 CM22-261_lowCpG_. Within nt 22-261 of *gag*, HIV-1 CM22-261_lowCpG_ has the same four CpG dinucleotides as wild type HIV-1 and 59 mutations, compared with HIV-1 CM 22-261 that has 22 CpG dinucleotides and 80 mutations (Fig. 6, Table 2). pHIV-1_NL4-3_, pHIV-1 CM22-261 and pHIV-1 CM22-261_lowCpG_ were transfected into HeLa cells and single round infectivity assays were performed. In contrast to HIV-1 CM22-261, HIV-1 CM22-261_lowCpG_ infectivity, Gag expression and virion production was similar to HIV-1_NL4-3_ (Fig. 7a, b). We also cloned pHIV-1 CM22-378_lowCpG_, which has four CpG dinucleotides and 79 mutations compared with the 30 CpG dinucleotides and 109 mutations in HIV-1 CM22-378 (Fig. 6, Table 2). In a single round infectivity assay, HIV-1 CM22-378_lowCpG_ also had similar levels of infectivity, intracellular Gag expression and virion production as HIV-1_NL4-3_ (Fig. 7c, d). While codon modification of nt 22-261 and 22-378 in *gag* substantially decreased the A-rich nucleotide bias of these regions, eliminating only the CpG dinucleotides in the codon modified sequence did not restore the A-rich bias for HIV-1 CM 22-261_lowCpG_ and HIV-1 CM22-378_lowCpG_ (Table 2). This supports the hypothesis that the decrease in infectivity for HIV-1 CM22-261 and HIV-1 CM22-378 is due to the introduced CpG dinucleotides and not changes in the A-rich nucleotide bias. In sum, changing the codons that introduced CpG dinucleotides in nt 22-261 or nt 22-378 back to the wild type HIV-1 codons while maintaining all of the other mutations in these regions restored infectious virus production.

Toll-like receptor 9 (TLR9) recognizes unmethylated CpG DNA motifs in endolysosomes within plasmacytoid dendritic cells, macrophages, and B cells [75]. Upon ligand binding, TLR9 signaling stimulates interferon alpha (IFN-α) production, which binds the interferon alpha and beta receptor (IFNAR) and induces interferon stimulated gene (ISG) expression via the JAK-STAT signaling pathway. To determine whether HeLa cells are responsive to CpG DNA or other TLR ligands, we tested a panel of ligands for TLR2, TLR3, TLR4, TLR5, TLR7, TLR8, TLR9 and TLR13 (Additional file 3). As positive controls, HeLa cells were infected with Sendai virus, which activates the cytoplasmic RNA sensors RIG-I and MDA5 (RIG-I-like receptors, RLR), or were treated with interferon beta (IFN-β), which also binds IFNAR. Both Sendai virus and IFN-β stimulated STAT1 phosphorylation and IFIT1 expression, which is an ISG. CpG DNA did not stimulate STAT1 phosphorylation or IFIT1 expression. The only TLR ligand that stimulated the cells was poly(I:C), which is structurally similar to double stranded RNA and can also signal via RLRs. Therefore, it is unlikely that the additional CpG dinucleotides in HIV-1 CM22-261 and HIV-1 CM22-378 inhibit viral replication via TLR9 or other TLRs in HeLa cells.

### Increased abundance of CpG dinucleotides is sufficient for inhibition of viral replication

**Fig. 8:**
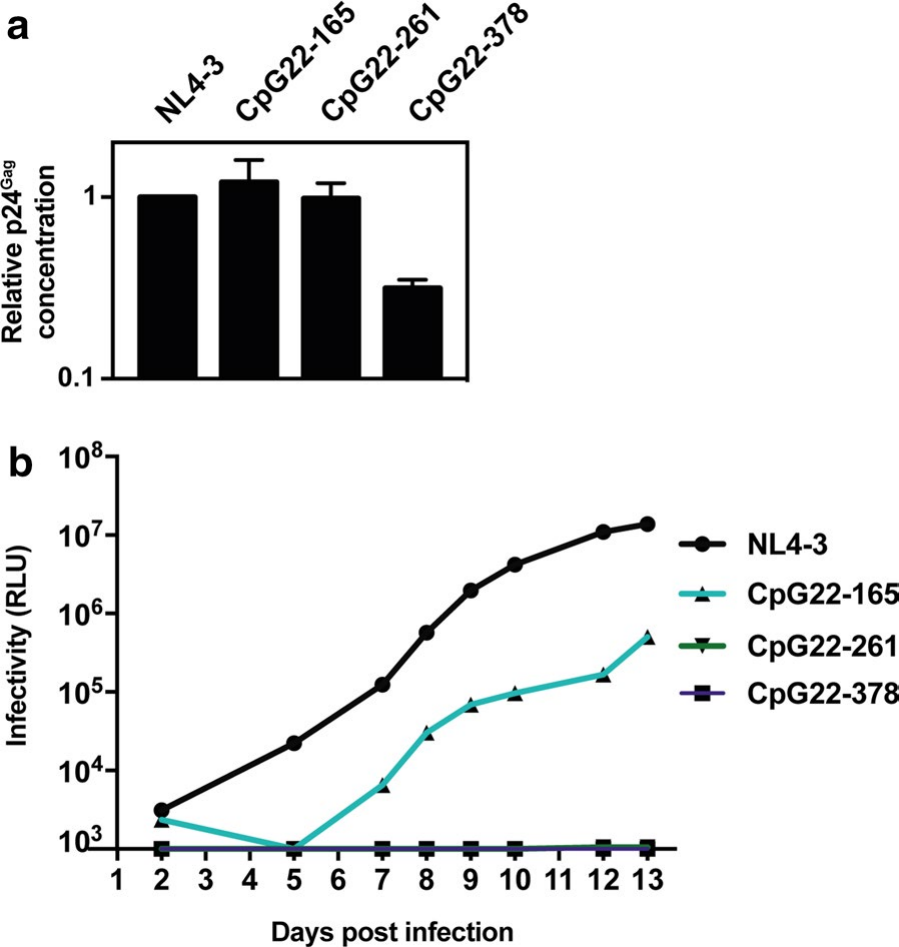
Introduction of CpG dinucleotides into *gag* inhibits HIV-1 replication in Jurkat cells. **a** The amount of HIV-1 CA (p24^Gag^) in supernatants from 293T cells transfected with pHIV-1_NL4-3_, pHIV-1 CpG22-165, pHIV-1 CpG22-261 or pHIV-1 CpG22-378 were quantified by p24^Gag^ ELISA. The bar chart is the average of three independent experiments normalized to HIV-1_NL4-3_. Error bars represent standard deviation. **b** Jurkat cells were infected with 25 ng of p24^Gag^ for each indicated virus. The amount of infectious virus present at each time point was measured in TZM-bl cells. This is representative of three independent experiments

To determine if increasing the abundance of CpG dinucleotides is sufficient to inhibit HIV-1 replication, we synthesized *gag* sequences in which only the codons that introduced CpG dinucleotides in the codon modified sequence were changed (Fig. 6). These were inserted into pHIV_NL4-3_ to produce pHIV-1 CpG22-165, pHIV-1 CpG22-261 and pHIV-1 CpG22-378. We then used a spreading infection assay in Jurkat cells to analyze the effect of the increased CpG abundance. 293T cells were transfected with each proviral construct to produce stocks of each virus and the abundance of p24^Gag^ was measured for each by ELISA. HIV-1 CpG22-165 and HIV-1 CpG22-261 produced similar amounts of virus to HIV-1_NL4-3_ while HIV-1 CpG22-378 virus production was decreased by ~ 70% (Fig. 8a). The viral inoculum was normalized so that Jurkat cells were infected with 25 ng of p24^Gag^ for each virus and replication was monitored over ~ 2 weeks. There are an additional 11 CpG dinucleotides in HIV-1 CpG22-165 and its replication was substantially reduced, with > 90% less infectivity at Day 13 compared to HIV-1_NL4-3_ (Fig. 8b). HIV-1 CpG22-261 has an additional 18 CpG dinucleotides and HIV-1 CpG22-378 has an additional 26 CpG dinucleotides. Neither of these viruses replicated in the Jurkat cells (Fig. 8b). Therefore, introducing CpG dinucleotides into HIV-1 *gag* is sufficient to strongly attenuate viral replication in Jurkat cells.

**Fig. 9:**
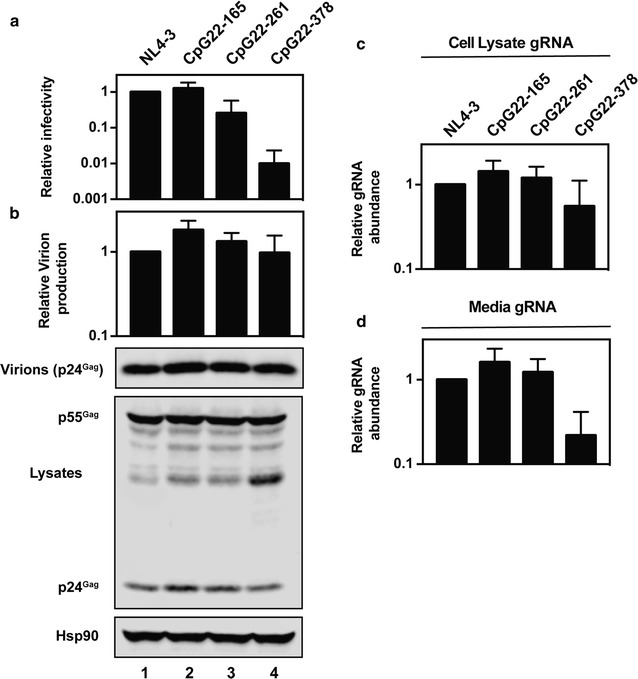
Introduction of CpG dinucleotides into *gag* inhibits infectious virus production in HeLa cells. HeLa cells were transfected with pHIV-1_NL4-3_, pHIV-1 CpG22-165, pHIV-1 CpG22-261 or pHIV-1 CpG22-378. **a** Culture supernatants were used to infect TZM-bl reporter cells to measure viral infectivity. The bar charts show the average values of four independent experiments normalized to the value obtained for HIV-1_NL4-3_. **b** Gag expression in the media and cell lysate was determined by quantitative immunoblotting. The bar charts show the average of three independent experiments normalized to HIV-1_NL4-3_. Error bars represent standard deviation. RNA was extracted from cell lysates and media (**c**, **d**) and gRNA abundance was quantified by qRT-PCR. **b**–**d** The bar charts show the average of three independent experiments normalized to HIV-1_NL4-3_. Error bars represent standard deviation

To analyze the steps of the HIV-1 life cycle that were inhibited by the CpG dinucleotides, HeLa cells were transfected with pHIV_NL4-3_, pHIV-1 CpG22-165, pHIV-1 CpG22-261 or pHIV-1 CpG22-378. ~ 48 h later, the media and cell lysates were harvested for infectivity, protein or RNA analysis. In this single cycle assay, HIV-1 CpG22-378 infectivity was decreased ~ 99% (Fig. 9a). While there was no change in Gag expression or virion production (Fig. 9b), HIV-1 CpG22-378 gRNA was decreased ~ 40% in the cell lysate and ~ 80% in the media (Fig. 9c, d). HIV-1 CpG22-261 had a ~ 75% decrease in infectivity with no difference in Gag expression, virion production or gRNA abundance (Fig. 9a–d). There was no decrease in infectious virus production for HIV-1 CpG 22-165. Overall, introducing 26 CpG dinucleotides into HIV_NL4-3_ decreased infectious virus production by ~ 99%, though this is a smaller attenuation than viruses containing CpG dinucleotides in the context of the codon modified sequence (Figs. 2, 4, 7).

One potential explanation for why HIV-1 containing CpG dinucleotides in a codon modified context produce less infectious virus in HeLa cells than HIV-1 in which only CpG dinucleotides have been added is that a nucleic acid binding protein could bind the CpG dinucleotide to mediate antiviral activity. If a protein does directly bind the CpG dinucleotide, its binding site may encompass more than just the CpG and the surrounding nucleotides may affect binding. Therefore, the five nucleotides 5′ and 3′ of the introduced CpG in HIV-1 CM22-378 and HIV-1 CpG22-378 were used to generate a 12 nt sequence. These were aligned and conserved nucleotides were identified using WebLogo [67]. Interestingly, the nucleotides surrounding the CpG in the codon modified sequence are more G/C-rich than in the wild type HIV-1 sequence (Additional file 4A and B).

## Discussion

Herein, we show that introducing CpG dinucleotides into the HIV-1 genome inhibits viral replication. When only 11 CpG dinucleotides are inserted into *gag* in the context of the codon modified sequence (HIV-1 CM22-165), there is a large decrease in infectivity without a substantial loss of gRNA abundance, Gag expression or virion production (Figs. 2, 3). When 18 or 26 CpG dinucleotides are added (HIV-1 CM22-261 and HIV-1 CM22-378, respectively), the intracellular gRNA stability and abundance is decreased which leads to reduced Gag expression and virion production (Figs. 2, 3, 4, 5). In addition, there is a defect in the pre-integration steps of the viral life cycle that is apparent when equivalent numbers of genomes are used to infect target cells (Fig. 3c). Thus, manipulating the CpG abundance in *gag* can impart both producer and target cell defects in replication. Determining whether these deficiencies are underpinned by a common mechanism such as shared host factors, or whether they are distinct from each other, will be an important direction of our future work.

Remarkably, none of the proposed *cis*-acting elements in nt 22-378 of *gag* appear to be necessary for infectious virus production in HeLa cells. We demonstrated this by comparing viruses that have nt 22-261 or nt 22-378 codon modified or deleted (Fig. 4). While codon modification of these regions inhibited HIV-1 infectivity, deletion of the same region did not substantially impair infectivity in a single round assay. Furthermore, removing the introduced CpG dinucleotides in HIV-1 CM22-261 and HIV-1 CM22-378 while maintaining either 59 or 79 other nucleotide changes, respectively, almost completely restored HIV-1 infectious virus production (Fig. 7). This supports the observation by Reil et al. [60] that the globular head of MA can be deleted in the context of Env with a truncated cytoplasmic domain without substantially impairing viral replication in the MT4 T cell line or virion production in HeLa cells.

One of the *cis*-acting elements proposed to be in the *gag* region that we have mutated is the gRNA packaging signal. While a relatively short sequence sufficient for packaging heterologous transcripts into virions has been identified for rous sarcoma virus and murine leukemia virus, delineation of the HIV-1 RNA sequence that is sufficient for packaging has been more controversial [7, 8]. The core packaging signal for HIV-1 is in the 5′ UTR; however, approximately 300 nt of the 5′ end of *gag* has been proposed to improve viral titre [7, 8]. We did not codon modify the first 21 nt of *gag* because this region is under purifying selection [11, 12] and NMR structures have shown that it base pairs with the U5 region of the 5′ UTR to form the dimer promoting conformation of the gRNA that is packaged into virions [10, 76]. However, the relative importance of the sequence in *gag* beyond the first 21 nt for packaging the full-length HIV-1 gRNA, as opposed to heterologous transcripts, is unclear. Mapping Gag binding sites on the gRNA in living cells showed that the RNA elements most frequently crosslinked to Gag were in the 5′ UTR and the RRE [77]. In the context of the full-length virus, our data indicates that nt 22-378 in *gag* appear to have only a small effect on viral infectivity when this region is deleted or mutated without adding CpG dinucleotides (Figs. 4, 7). The requirement for this region for gRNA packaging may be different in the context of lentiviral vector genomes, which contain only a small portion of the HIV-1 gRNA [78].

In principle, codon usage changes in *gag* could affect gRNA translation [79]. However, the translation efficiency of a codon optimized *gag* mRNA is only ~ 1.6-fold higher than wild type *gag* mRNA that contains theoretically suboptimal codon usage [80]. Our data indicate that gRNA translation efficiency is not substantially affected by the changes in codon usage for HIV-1 CM22-261 since Gag expression (Fig. 2c) and intracellular gRNA abundance (Figs. 3c, 5a) were both decreased by ~ 90%. Changing the RNA sequence could also affect the secondary or tertiary structure of the gRNA. While the amount of RNA structure in the MA region of *gag* is much lower than that of the 5′ UTR [32], we cannot exclude that the synonymous mutations in *gag* have not altered nearby structures. The known structures in the nucleotides that we have mutated are the IRES [29, 53, 81, 82] and the region that base pairs with the 3′ end of the genome [33, 35]. In the context of single cycle infectivity assays in HeLa cells (Fig. 4) and replication in MT4 cells [60], neither the IRES nor circularization of the HIV-1 gRNA appear to be necessary because the region containing these elements can be deleted. However, it is possible that the phenotype for mutating an RNA structure may not be the same as deleting it [83] and these structures could be necessary under conditions not tested in this study, such as cellular stress or the innate immune response [6].

Two previous reports have shown that introducing synonymous mutations into *gag* or *pol* attenuates HIV-1 replication [54, 84]. Martrus et al. [84] introduced codon pairs into *gag* or *pol* that are underrepresented relative to human mRNAs, which strongly inhibited viral replication. However, codon pair bias in RNA viruses has recently been shown to be due CpG and UpA dinucleotide suppression and codon pair deoptimization increases CpG abundance [74, 85]. While not discussed in their study, the synonymous mutations that Martrus et al. introduced increased the number of CpG dinucleotides in *gag* from 15 to 118. Therefore, we hypothesize that the decrease in viral replication observed in this study is at least partially a result of increasing the CpG frequency instead of codon pair deoptimization. To analyze the role of the A-rich RNA sequence for HIV-1 replication, Keating et al. [54] codon modified regions within *gag* and *pol* by increasing the number of C- and G-rich codons, which also inhibited HIV-1 replication. phGag-Pol [86] was used as the source of codon modified sequence and this has a large increase in CpG dinucleotide abundance compared with the wild type sequence. These additional CpG dinucleotides could be responsible for viral attenuation instead of altering the A-rich codon bias. Supporting this hypothesis, Klaver et al. [87] used phylogeny-instructed mutagenesis to increase or decrease the A-rich codon bias in an ~ 500 nt region of *pol*. In this study, the CpG dinucleotide abundance was decreased by one in the A-Max mutant virus and only increased by four in the A-Min mutant virus. Substantially increasing or decreasing the number of A nucleotides did not affect HIV-1 replication, demonstrating that it is important to avoid introducing suppressed dinucleotides such as CpG in mutagenesis studies analyzing the functional relevance of nucleotide or codon bias.

While CpG dinucleotides appear to be under negative selection in HIV-1 [36, 40, 44, 55–57], the specific selection pressure has been unclear. There are at least four potential causes for the suppressed abundance of CpG dinucleotides in HIV-1. First, this could be due to a mutational bias caused by cytosines in a CpG context in the proviral DNA becoming methylated and then undergoing rapid spontaneous deamination. However, we have shown that increasing the abundance of CpG dinucleotides inhibits viral replication, even in a single round assay (Figs. 1, 2, 8, 9). Second, CpG methylation-induced transcriptional silencing could inhibit HIV-1 gene expression [36, 40, 44, 55–57, 88]. However, this cannot cause the inhibition in the single cycle infectivity assays because the transfected proviral plasmids were amplified in bacteria, which does not methylate CpG dinucleotides. Importantly, when an unmethylated plasmid is transfected into mammalian cells, the CpG dinucleotides are not methylated [89–91]. Third, unmethylated CpGs in the DNA could be recognized by TLR9 [88, 92]. This pattern recognition receptor is expressed in plasmacytoid dendritic cells, macrophages, and B cells [75] and therefore is unlikely to be expressed in HeLa cells. We confirmed that HeLa cells do not induce STAT1 phosphorylation or IFIT1 expression in response to CpG DNA (Additional file 3). In addition, IFN-α does not inhibit wild type HIV-1 infectious virus production in HeLa cells at most concentrations (10–1000 U/ml) and only has moderate inhibition at very high concentrations (10,000 U/ml) [93]. Therefore, the inhibition that we observe in response to introducing CpG dinucleotides into HIV-1 is unlikely to be due to the production of type I interferon.

The fourth possibility is that CpG dinucleotides in the viral RNA could induce an antiviral response that restricts HIV-1 replication. The frequency of CpG dinucleotides is suppressed in many RNA viruses that do not have a DNA intermediate [57, 92, 94–96], indicating that CpG DNA methylation or activation of TLR9 cannot be responsible for CpG suppression in all viruses. Introduction of CpG dinucleotides into picornaviruses or influenza A virus inhibits viral replication [71–74]. It is unclear how CpG dinucleotides restrict RNA virus replication but, for the picornavirus echovirus 7, it is not due to stimulating the interferon pathway, PKR, conventional pattern recognition receptors or altering the translation efficiency of viral proteins [71, 74]. It has been hypothesized that there is an innate immune sensor that detects CpG dinucleotides in viral RNA and leads to the inhibition of viral replication, though the molecular details are unknown [71, 73, 74, 85, 92, 97–99]. We favor the hypothesis that the proposed active restriction pathway targeting CpG dinucleotides in RNA viruses inhibits HIV-1 with an increased CpG abundance. When 26 CpG dinucleotides were added within nt 22-378 of *gag* (HIV-1 CpG22-378), which is < 5% of the HIV-1 gRNA, viral replication was inhibited in Jurkat cells (Fig. 8) and infectivity was decreased by ~ 99% in a single round assay (Fig. 9). This indicates that the CpG dinucleotides induce a potent restriction.

While this manuscript was under review, Takata et al. [100] reported that introducing CpG dinucleotides into *env* inhibited HIV-1 replication by decreasing the abundance of cytoplasmic gRNA, Gag expression, Env expression and infectious virus production. They also demonstrated that depleting the cellular RNA binding protein ZAP rescues replication of HIV-1 with increased CpG abundance and ZAP directly binds HIV-1 RNA regions containing CpG dinucleotides. This indicates that ZAP restricts replication of HIV-1 containing increased CpG abundance, though it is unclear how ZAP promotes viral RNA degradation. Interestingly, we and others have shown that Gag is efficiently expressed from mammalian expression vectors that encode codon-optimized *gag* or *gag*-*pol* cDNAs containing large numbers of CpG dinucleotides [61, 68, 86, 101, 102]. Therefore, it appears that CpG dinucleotides in the context of full length HIV-1 is more deleterious for protein expression than CpGs in the context of mammalian expression vectors. How the specific context of CpG dinucleotides affects ZAP binding to RNA or modulates its activity will be an exciting area of future research.

## Conclusions

The HIV-1 RNA sequence contains specific nucleotide features such as a low abundance of CpG dinucleotides. Our data shows that introducing CpG dinucleotides into HIV-1 inhibits viral replication by affecting multiple steps of the life cycle. This provides a functional explanation for why CpG dinucleotides are suppressed in HIV-1 and we speculate this dinucleotide is under negative selection to avoid an active restriction system that may require ZAP. Understanding how this restriction system inhibits replication of HIV-1 with increased CpG abundance may provide insight into how other RNA viruses, such as picornaviruses and influenza A virus, are attenuated when CpG dinucleotides are introduced.

## Additional files

**Additional file 1.** Codon modification of nucleotides 22-261 in gag inhibits viral replication in SupT1 cells. SupT1 cells were infected with 10 ng of p24Gag for each indicated virus. The amount of infectious virus present at each time point was measured in TZM-bl cells. This is representative of three independent experiments.

**Additional file 2.** Codon modification of nucleotides 22-261 in gag decreases total HIV-1 RNA abundance. The RNA that was extracted from cell lysates as described for Fig. 3a was quantified for total HIV-1 RNA by qRT-PCR.

**Additional file 3.** CpG DNA does not stimulate STAT1 phosphorylation or ISG expression. HeLa cells were stimulated with TLR ligands for 5 h. TLR3 was targeted with 0.1, 1, 10 μg/ml polyIC, TLR7 with 0.3, 3, 30 nM Gardiquimod (Gard), TLR8/7 with 0.3, 3, 30 nM CL075, TLR7/8 with 0.01, 0.1, 1 μg/ml R848, TLR9 with 0.01, 0.1, 1 μg/ml CpG DNA, TLR13 with 0.01, 0.1, 1 μg/ml 23S ribosomal RNA (rRNA). TLR 4, 2 and 5 were targeted with 0.1 μg/ml LPS, Pam3Cys (P3C) or Flagellin (FliC), respectively. As controls for pattern recognition receptor signaling and JAK-STAT signaling, cells were infected with 50 HAU/ml Sendai virus (SeV) for 5 h or stimulated with 0.01 μg/ml IFN-β for 1 h, respectively. Activation of IFN signaling was monitored by western immunoblotting against phosphorylated STAT1 (pSTAT1) or expression of the ISG IFIT1. Actin was used as a loading control. (* Denotes the molecular weight marker).

**Additional file 4.** The CpG dinucleotide in the codon modified sequence is in a G/C-rich context. The sequence of the five nucleotides 5′ and 3′ to the CpG dinucleotides introduced into HIV-1 CM22-387 and HIV-1 CpG22-387 were aligned and a graphical representation of the sequence conservation was generated by WebLogo.
