# Supplementary figures and images for: Increasing the CpG dinucleotide abundance in the HIV-1 genomic RNA inhibits viral replication
Source: Retrovirology. 2017 Nov 9;14:49. doi: 10.1186/s12977-017-0374-1 (PMC5679385; doi:10.1186/s12977-017-0374-1)

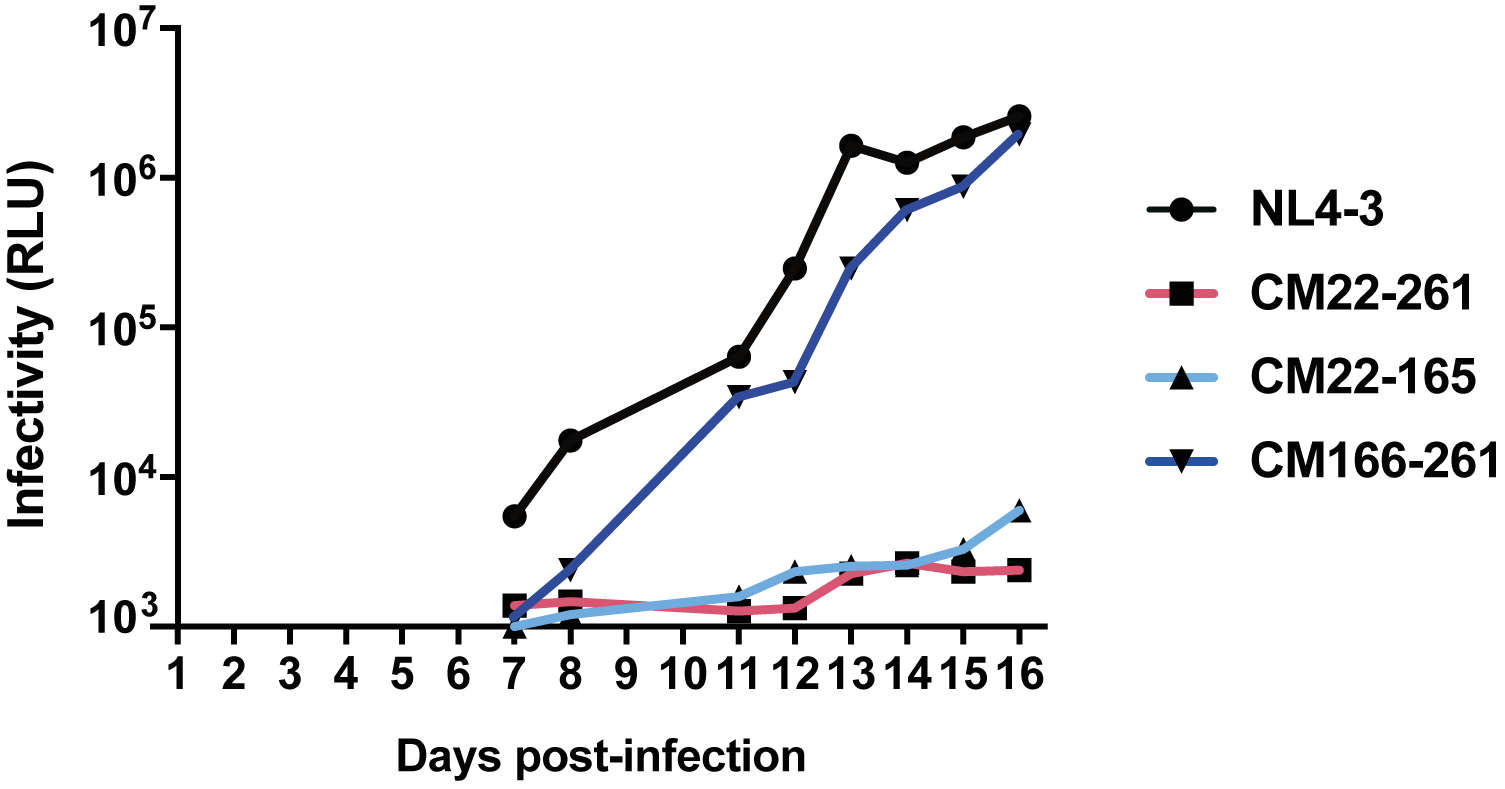

Supplement: Supplementary file 1 — Additional file 1. Codon modification of nucleotides 22-261 in gag inhibits viral replication in SupT1 cells. SupT1 cells were infected with 10 ng of p24Gag for each indicated virus. The amount of infectious virus present at each time point was measured in TZM-bl cells. This is representative of three independent experiments. [file 12977_2017_374_MOESM1_ESM.tif]

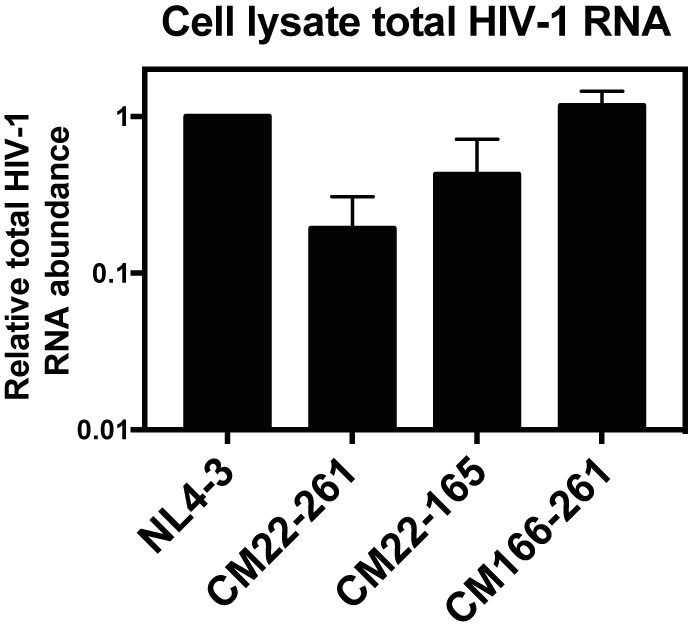

Supplement: Supplementary file 2 — Additional file 2. Codon modification of nucleotides 22-261 in gag decreases total HIV-1 RNA abundance. The RNA that was extracted from cell lysates as described for Fig. 3a was quantified for total HIV-1 RNA by qRT-PCR. [file 12977_2017_374_MOESM2_ESM.tif]

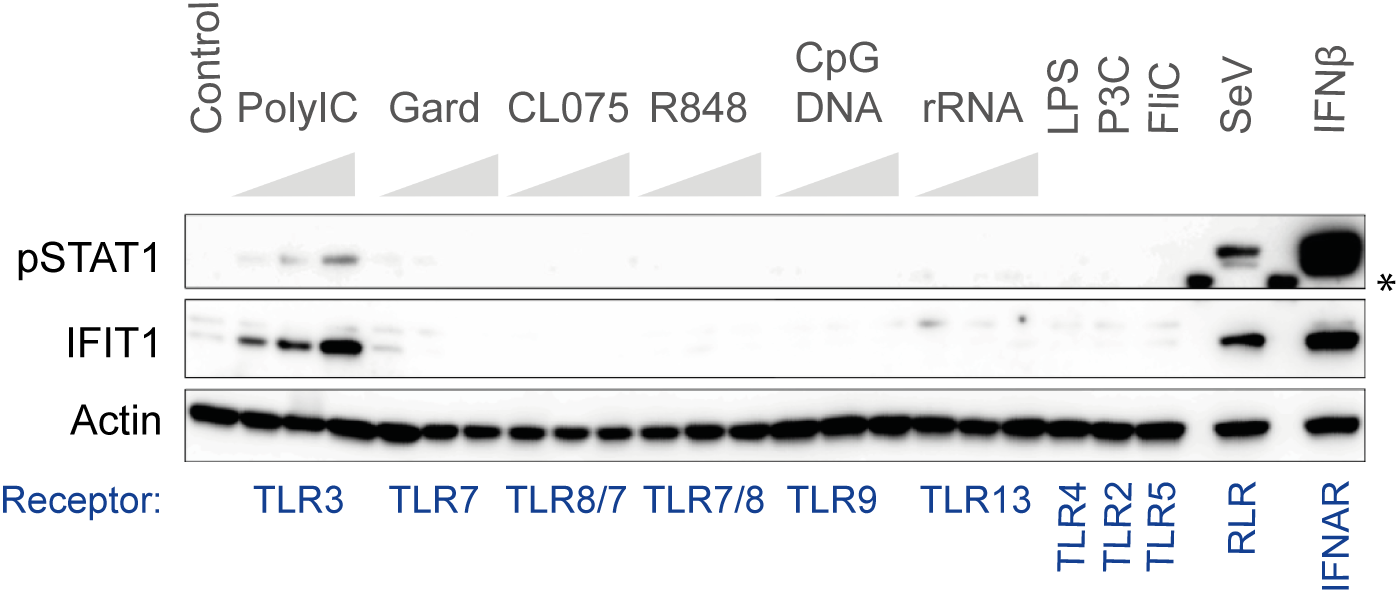

Supplement: Supplementary file 3 — Additional file 3. CpG DNA does not stimulate STAT1 phosphorylation or ISG expression. HeLa cells were stimulated with TLR ligands for 5 h. TLR3 was targeted with 0.1, 1, 10 μg/ml polyIC, TLR7 with 0.3, 3, 30 nM Gardiquimod (Gard), TLR8/7 with 0.3, 3, 30 nM CL075, TLR7/8 with 0.01, 0.1, 1 μg/ml R848, TLR9 with 0.01, 0.1, 1 μg/ml CpG DNA, TLR13 with 0.01, 0.1, 1 μg/ml 23S ribosomal RNA (rRNA). TLR 4, 2 and 5 were targeted with 0.1 μg/ml LPS, Pam3Cys (P3C) or Flagellin (FliC), respectively. As controls for pattern recognition receptor signaling and JAK-STAT signaling, cells were infected with 50 HAU/ml Sendai virus (SeV) for 5 h or stimulated with 0.01 μg/ml IFN-β for 1 h, respectively. Activation of IFN signaling was monitored by western immunoblotting against phosphorylated STAT1 (pSTAT1) or expression of the ISG IFIT1. Actin was used as a loading control. (* Denotes the molecular weight marker). [file 12977_2017_374_MOESM3_ESM.tif]

### A) Codon modified context

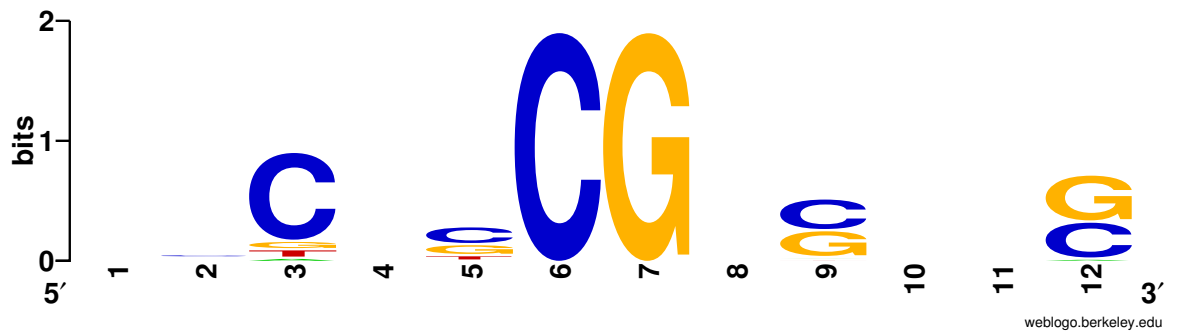

### B) Wild type HIV-1 context

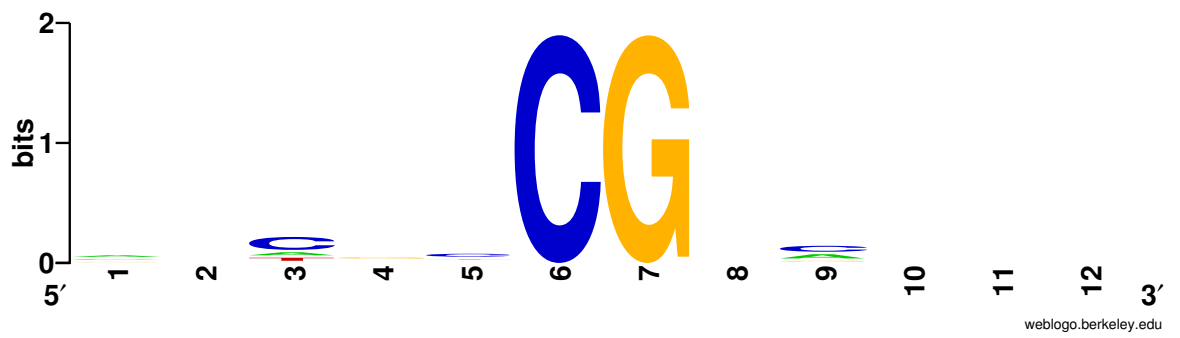

Supplement: Supplementary file 4 — Additional file 4. The CpG dinucleotide in the codon modified sequence is in a G/C-rich context. The sequence of the five nucleotides 5′ and 3′ to the CpG dinucleotides introduced into HIV-1 CM22-387 and HIV-1 CpG22-387 were aligned and a graphical representation of the sequence conservation was generated by WebLogo. [file 12977_2017_374_MOESM4_ESM.pdf]
